# *Helicobacter pylori* CagA disrupts pancreatic epithelial barrier integrity

**DOI:** 10.3389/fcell.2025.1688326

**Published:** 2025-11-18

**Authors:** Anais Romero-Fabela, Angelica Silva-Olivares, Melisa Chacon, Erika P. Rendon-Huerta, Abigail Betanzos

**Affiliations:** 1 Departamento de Infectómica y Patogénesis Molecular, Centro de Investigación y de Estudios Avanzados (Cinvestav), Ciudad de Mexico, Mexico; 2 Departamento de Biología Celular y Tisular, Laboratorio de Inmunobiología, Facultad de Medicina, Universidad Nacional Autónoma de México (UNAM), Ciudad de Mexico, Mexico

**Keywords:** BXPC-3 cells, apical junctional complex, tight junction, actin cytoskeleton, migration, IL-8

## Abstract

**Introduction:**

The oncoprotein CagA is the major virulence factor of *Helicobacter pylori* and plays a central role in the development of gastric disorders. *H. pylori* CagA^+^ infection has been implicated with an increased risk of developing extragastric diseases such as pancreatic cancer; however, the mechanisms remain unclear.

**Methods:**

In this study, BxPC-3 pancreatic epithelial cells were infected with *H. pylori* bacterial strains CagA^+^ (26695 and PMSS1) or CagA^−^ (Tx30a).

**Results:**

Infection with CagA^+^ strains resulted in a significant disruption of epithelial barrier integrity, as demonstrated by transepithelial electrical resistance assays. Western blot and immunofluorescence analyses revealed altered expression and cytoplasmic relocalization of key apical junctional complex proteins. Meanwhile, claudin-4 and occludin levels increased, that of ZO-1 remained unchanged, and those of E-cadherin and β-catenin decreased. Likewise, cytoskeletal rearrangements were observed, particularly the loss of the actin apical ring and mislocalization of actin-binding proteins, such as cortactin and vinculin, at the cell borders. The loss of barrier integrity and cytoskeletal alterations were associated with morphological changes and increased cell motility, as demonstrated by wound healing assays. In addition, infection was accompanied by an increase in IL- 8 secretion.

**Discussion:**

These findings suggest that CagA alters the pancreatic epithelial cells’ functions. Therefore, CagA represents an undescribed risk factor in the pathogenesis of *H. pylori*-associated pancreatic illness.

## 1 Introduction

The global prevalence of *Helicobacter pylori* is estimated at 44.35%, with a higher rate observed in developing countries (50.8%) than in developed nations (34.7%) (Zamani et al., 2018). Gastric colonization by *H. pylori* is associated with a variety of upper gastrointestinal disorders, including chronic gastritis, peptic ulcer disease, gastric mucosa-associated lymphoid tissue lymphoma, and gastric cancer (Kusters et al., 2006).

One of the key pathological mechanisms in *H. pylori*-associated inflammation and neoplastic transformation is the disruption of the epithelial barrier, particularly the opening of the apical junctional complex (AJC), including tight junctions (TJs) and adherens junctions (AJs). TJs composed of proteins such as ZO-1, JAM, occludin, and claudins delineate the apical basolateral boundary of polarized epithelial cells and form a highly selective barrier to prevent paracellular leakage of ions and macromolecules (Wroblewski and Peek, 2011). Early studies demonstrated that the *H. pylori* oncoprotein CagA interacts with ZO-1 and JAM, leading to ectopic assembly of TJ components at bacterial adhesion sites and thereby altering the structure and function of the AJC (Amieva et al., 2003). Further investigations revealed that the ectopic expression of CagA disrupts cell-to-cell junctions, a process dependent on its phosphorylation status and specific structural domains (Takahashi-Kanemitsu et al., 2020). Gastric carcinogenesis is also associated with altered expression of several claudins in the gastric mucosa. *De novo* expressions of claudin-4, -6, -7, and -9 have been reported as particularly relevant (Iravani et al., 2013). Notably, overexpressions of claudin-6, -7, and -9 are strongly correlated with enhanced tumorigenic properties, including increased cellular invasiveness and metastatic potential (Zavala-Zendejas et al., 2011).

CagA is a 128- to 145-kDa protein translocated into host cells via the type IV secretion system, where it targets various host cell kinases (Zeng et al., 2020). Structurally, CagA consists of a conserved N-terminal domain and a highly variable, intrinsically disordered C-terminal region (Takahashi-Kanemitsu et al., 2020). This region contains a variable number of Glu-Pro-Ile-Tyr-Ala (EPIYA) phosphorylation motifs (Hayashi et al., 2012). Upon delivery into host cells, CagA localizes to the plasma membrane and is phosphorylated by host tyrosine kinases such as c-Src and c-Abl (Backert et al., 2016). The phosphorylated form of CagA (CagA^PY^) interacts with proteins such as SHP2, Csk, and Crk, triggering activation of the ERK/MAPK signaling pathway. This leads to dysregulated epithelial gene expression and the characteristic “hummingbird phenotype” (Wang et al., 2023). Eventually, phosphorylated CagA inactivates Src kinase through a negative feedback loop. This inactivation disrupts the function of proteins essential for actin cytoskeleton regulation, including cortactin, ezrin, and vinculin (Sharafutdinov et al., 2021). In its non-phosphorylated state, CagA interacts with E-cadherin and disrupts AJs in polarized gastric epithelial cells. This leads to cytoplasmic and nuclear accumulation of β-catenin, activation of Wnt signaling pathways, and subsequent malignant transformation and cell migration (Zheng et al., 2021). CagA also induces nuclear translocation of the NF-κB p65 subunit, thereby promoting transcriptional activation and secretion of the pro-inflammatory cytokine IL-8 (Tohidpour, 2016).

Emerging evidence suggests that *H. pylori* infection may also have extragastric effects, potentially contributing to neurodegenerative, metabolic, and cardiovascular conditions, along with hepatobiliary, colorectal, and pancreatic diseases, such as pancreatic cancer, autoimmune pancreatitis, metabolic syndrome, and diabetes *mellitus* (M. Bulajic and Löhr JM, 2014; Rabelo-gonçalves et al., 2015). For example, Stolzenberg-Solomon et al. (2001) found that individuals seropositive for *H. pylori* had a higher risk of developing pancreatic cancer compared to seronegative individuals, with an odds ratio (OR) of 2.01 (95% CI: 1.09–3.70) versus an OR of 1.87 (95% CI: 1.05–3.34) (Stolzenberg-solomon et al., 2001). Similarly, Ai et al. (2015) reported an increased risk of pancreatic cancer in patients infected with *H. pylori*, particularly those seropositive for CagA (Ai et al., 2015). These findings suggest a potential role of *H. pylori,* especially CagA^+^ strains, in the pathogenesis of pancreatic cancer.

Moreover, the presence of *H. pylori* in the pancreas of gerbils demonstrated that the pancreatic epithelium was molecularly affected. In particular, alterations were observed in several TJ and AJ proteins, including claudin-1, claudin-4, occludin, ZO-1, E-cadherin, and β-catenin, along with a rearrangement of the actin cytoskeleton. These structural changes were accompanied by altered distribution patterns of insulin and glucagon, along with evidence of systemic inflammation (Hurtado-Monzón et al., 2024).

Based on these observations, the present study aimed to investigate the effects of the *H. pylori* virulence factor CagA on the human pancreatic cancer cell line BxPC-3. In particular, we examined the alterations induced by infection with *H. pylori* strains either expressing or lacking CagA in intercellular junction proteins, the organization of the actin cytoskeleton and its associated regulatory proteins, as well as cell migration and the expression of the pro-inflammatory cytokine IL-8.

## 2 Materials and methods

### 2.1 Culture of *Helicobacter pylori* strains

The *H. pylori* CagA^+^ strains 26695 (ATCC 700392, Manassas, VA, United States) and PMSS1 and the strain Tx30a CagA^−^ (ATCC 51932, Manassas, VA, United States) (Pillinger et al., 2007; Jang et al., 2017; Kurniasari et al., 2023) were cultured on Casman agar plates supplemented with 5% fresh defibrinated sheep blood. Cultures were incubated at 37 °C under microaerophilic conditions (in a CO_2_ incubator with 85% humidity) to maintain optimal bacterial growth. Fresh cultures were initiated from glycerol stocks and subcultured every 2–3 days to maintain viability and virulence.

### 2.2 Culture of epithelial cells

The human pancreatic epithelial cell line BxPC-3 (CRL-1687), derived from a ductal adenocarcinoma (Weddle et al., 2001), was cultured in RPMI-1640 medium (Gibco, Miami, FL, United States) supplemented with 10% fetal bovine serum, 0.25 μg/mL amphotericin B, 100 U/mL penicillin, and 0.1 mg/mL streptomycin. Cells were maintained at 37 °C in a humidified atmosphere containing 5% CO_2_.

### 2.3 Bacterial infection of epithelial cells

BxPC-3 cells were incubated with *H. pylori* strains at a multiplicity of infection (MOI) of 1:100 for 3, 6, 9, 12, and 24 h in antibiotic- and antimycotic-free culture medium. Uninfected control cells (mock) were treated with an equivalent volume of phosphate-buffered saline (PBS: 137 mM NaCl, 2.7 mM KCl, 10 mM Na_2_HPO_4_, and 1.8 mM KH_2_PO_4_) under the same conditions.

### 2.4 SDS-PAGE and immunoblot analysis

Following infection, cells were harvested using a cell scraper in RIPA buffer (10 mM Tris-HCl, 1 mM EDTA, 140 mM NaCl, 0.5 mM EGTA, 1% Triton X-100, 0.1% sodium deoxycholate, 0.1% SDS, and 1× Complete™ protease inhibitor cocktail [Roche, Grenzach-Wyhlen, Germany]). Cell lysates were sonicated three times for 10 s and centrifuged at 14,000 rpm for 15 min to remove insoluble debris. Proteins were separated on 8%–12% SDS-PAGE and transferred onto nitrocellulose membranes for Western blot analysis. In parallel, some gels were stained with Coomassie Brilliant Blue 0.1% to assess total protein loading.

Membranes were blocked with 10% non-fat dry milk in Tris-buffered saline with 0.1% Tween-20 (TBST) for 2 h at room temperature (RT). The following primary antibodies were used: monoclonal anti-CagA (1:300; Santa Cruz Biotechnology; sc-28368, Dallas, TX, United States), monoclonal anti-phosphotyrosine PY99 (1:300; Santa Cruz Biotechnology; sc-7020, Dallas, TX, United States), monoclonal anti-claudin-4 (1:500; Life Technologies; 32-9400, Carlsbad, CA, United States), polyclonal anti-occludin (1:500; Invitrogen; 71-1500, Carlsbad, CA, United States), polyclonal anti-ZO-1 (1:500; Invitrogen; 61-7300, Carlsbad, CA, United States), monoclonal anti-E-cadherin (1:1,000; Santa Cruz Biotechnology; sc-59778, Dallas, TX, United States), monoclonal anti-β-catenin (1:1,000; Santa Cruz Biotechnology; sc-376959, Dallas, TX, United States), monoclonal anti-actin (1:1000; Santa Cruz Biotechnology; sc-47778, Dallas, TX, United States), monoclonal anti-cortactin (1:500; Sysy antibodies; 313111), monoclonal anti-vinculin (1:500; Santa Cruz Biotechnology; sc-25336, Dallas, TX, United States), and polyclonal anti-GAPDH (1:5,000; Invitrogen; PA5-85074, Carlsbad, CA, United States). HRP-conjugated secondary antibodies (anti-mouse or anti-rabbit; 1:3,000; Invitrogen, Carlsbad, CA, United States) were used, and bands were visualized using the SuperSignal™ West Femto chemiluminescent substrate (ThermoFisher Scientific, Waltham, MA, United States). Band intensities were quantified by densitometry using ImageJ software and normalized to GAPDH levels.

### 2.5 Immunofluorescence assays

BxPC-3 cells were cultured until reaching confluence and fixed with 4% paraformaldehyde (PFA) for 30 min at RT. Following fixation, cells were permeabilized with 0.2% Triton X-100 for 15 min and subsequently blocked with 1% bovine serum albumin (BSA) for 2 h at RT. Cells were incubated overnight (ON) at 4 °C with the following primary antibodies: anti-CagA (1:50), anti-claudin-4 (1:50), anti-ZO-1 (1:50), anti-E-cadherin (1:50), anti-β-catenin (1:100), anti-cortactin (1:200), and anti-vinculin (1:100). After washing with PBS, cells were incubated for 2 h at RT in the dark with Alexa Fluor 488-conjugated secondary antibodies (anti-rabbit or anti-mouse; 1:100; Invitrogen, Carlsbad, CA, United States). F-actin was visualized using phalloidin conjugated to Alexa Fluor 488 (1:400; Invitrogen, Carlsbad, CA, United States), and nuclei were stained with 2.5 μg/mL DAPI for 10 min. After additional PBS washes, VECTASHIELD was used as the mounting medium. Fluorescent images were acquired using a Carl Zeiss LSM 700 confocal microscope and processed using ZEN 2009 Light Edition software (Zeiss).

For stress fiber quantification, TIFF images captured at ×63 magnification were converted to 8-bit grayscale and calibrated to optical density (OD) values using ImageJ. The foreground and background colors were inverted. The ImageJ threshold tool was applied individually to determine an OD cutoff that encompassed most visible F-actin bundles while excluding background fluorescence. For each peripheral extension, a line selection perpendicular to the extension axis was drawn. The ImageJ plot profile function was used to obtain average pixel intensities at multiple points along the line. Then, the density ratio was calculated as the length along the line occupied by pixels with OD values above the threshold divided by the total line length. The peripheral stress fiber density entering a cell extension per cell was determined by averaging the density ratios across all extensions within that cell. For each experimental condition, 100 cells were analyzed, using the nuclei as a reference point (Peacock et al., 2007).

### 2.6 Transepithelial electrical resistance (TEER)

BxPC-3 pancreatic cells were cultured on Transwell filters (0.4 µm pore size; Corning, Glendale, AZ, United States) until stable TEER values of approximately 300 Ω cm^2^ were achieved. Monolayers were then infected apically with *H. pylori* strains 26695, PMSS1, or Tx30a for 24 and 48 h. TEER was measured using an EVOM epithelial voltmeter (World Precision Instruments, Sarasota, FL, United States). TEER values were corrected by subtracting the resistance of cell-free filters and normalized to the baseline value recorded prior to infection.

### 2.7 Dextran flux

BxPC-3 cells were seeded on Transwell filters (0.4 µm pore size; Corning, Glendale, AZ, United States) pre-coated with 0.8% gelatin and cultured for 72 h. Subsequently, 100 µg of 3–5 kDa FITC–dextran (Sigma-Aldrich, San Louis, MO, United States) was added to the upper chamber. After 1 h of incubation at 37 °C with gentle shaking in the dark, 100-µL samples were collected from the basal chamber. The amount of diffused fluorescent tracer was quantified using a fluorimeter (excitation wavelength: 547 nm; emission wavelength: 572 nm). Fluorescence values were converted to FITC–dextran concentrations using a standard calibration curve (Astre et al., 2024).

### 2.8 Wound healing assay

BxPC-3 cells were seeded to confluence in 24-well tissue culture plates. A mechanical wound was generated by scratching the monolayer with a pipette tip. Cell debris was removed by washing twice with fresh RPMI-1640 medium. Cells were then infected with *H. pylori* for 24 and 48 h. Wound closure was assessed by optical microscopy. For quantification, three representative images of the wound area were analyzed using ImageJ software (version 1.52q).

### 2.9 ELISA for detection of IL-8

Pancreatic cells were infected with the *H. pylori* strains. After 24 h, the culture supernatants were collected, and IL-8 secretion was analyzed using a commercially available ELISA kit (Abcam, Cambridge, United Kingdom). Pre-coated 96-well plates were loaded with 100 µL of either IL-8 standards or test samples and incubated at RT for 2 h. Control wells received only the sample dilution buffer. Following incubation, plates were washed three times with washing buffer and incubated for 60 min at RT with 100 µL of a biotin-labeled anti-IL-8 antibody. After an additional three washes, 100 µL of HRP-conjugated streptavidin was added, and the plates were incubated for 30 min at RT. Plates were then washed five times and incubated in the dark for 15 min with 100 µL of the TMB substrate solution. The reaction was stopped by adding 100 µL of stop solution, and the absorbance was immediately measured at 450 nm using an EPOCH microplate reader (BioTek, Santa Clara, CA, United States).

### 2.10 Statistical data analysis

Every experiment was performed in triplicate, and statistical significance was calculated using GraphPad Prism statistical software (version 8.0). Data were evaluated via one-way ANOVA followed by Student’s t-test with a Bonferroni correction. Significant difference was defined by (*) p < 0.05, (**) p < 0.01, (***) p < 0.001, and (****) p < 0.0001.

## 3 Results

### 3.1 CagA is translocated into pancreatic cells

**FIGURE 1:**
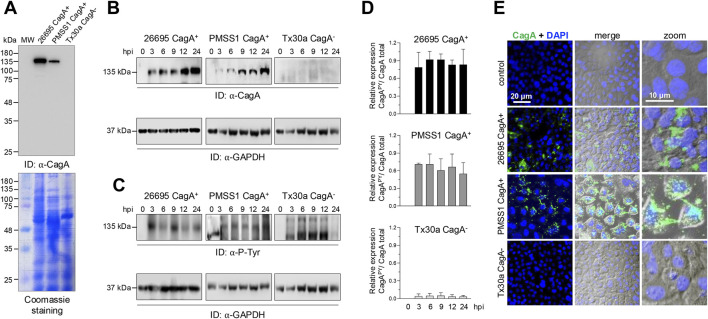
Detection and localization of CagA in BxPC-3 cells. **(A)** Expression of CagA in different *Helicobacter pylori* strains. Bacterial protein extracts were separated using SDS-PAGE and analyzed by Western blot using the anti-CagA (α-CagA) antibody. Parallel gels were stained with Coomassie Brilliant Blue to assess overall protein loading. MW: molecular weight markers (kDa). **(B,C)** Western blot analysis of BxPC-3 cell lysates after infection with *Helicobacter pylori* strains at several hours post-infection (hpi). Blots were probed with α-CagA **(B)** or anti-phosphotyrosine (α-P-Tyr) antibodies **(C)** to detect total and phosphorylated CagA, respectively. GAPDH was used as a loading control. Arrowheads indicate the immunodetected (ID) protein bands. **(D)** Densitometric analysis was performed based on the 135-kDa phosphorylated protein **(C)** and normalized to the total amount of CagA **(B)** for each strain. **(E)** Subcellular localization of CagA in BxPC-3 cells infected with *Helicobacter pylori* strains for 24 hpi. Cells were stained with anti-CagA antibody (green). Nuclei were counterstained with DAPI (blue), and phase-contrast images are shown in grayscale. Selected regions are shown at higher magnification in the right panels. Bar = 20 μm.

To investigate the relevance of CagA in pancreatic cells during *H. pylori* infection, we infected BxPC-3 cells with bacterial strains either carrying or lacking the chromosomal pathogenicity island *cag*PAI. In particular, strains 26695 and PMSS1 express CagA, while the strain Tx30a does not (Pillinger et al., 2007; Jang et al., 2017; Kurniasari et al., 2023). The presence or absence of CagA was confirmed by Western blot of strains 26695, PMSS1, and Tx30A cell lysates, and to confirm the equal loading of samples, a parallel gel was stained with Coomassie Brilliant blue; the result corroborated the expression of CagA only in 26695 and PMSS1 strains, but it also showed a difference in the CagA protein concentration between both strains (Figure 1A). To significantly contribute with the epithelial injury, CagA must be internalized by host cells. The amount of CagA in BxPC-3 cells was time-dependent and probably concentration-dependent (Figure 1B). This has already been demonstrated in gastric AGS cells through Western blot analysis, which showed a time-dependent increase in CagA expression levels sustained throughout the infection process (Sain et al., 2025). In addition, the variability in the number of *cagA* copies differs between strains and is proportional to the levels of virulence phenotypes, such as cell elongation and IL-8 induction (Jang et al., 2017).

Not all of the CagA protein is internalized; some remains associated with the bacteria adhered to the epithelial membrane. To confirm internalization and phosphorylation, a 135-kDa CagA^PY^ band was detected exclusively in lysates of BxPC-3 cells infected with CagA^+^ strains (Figure 1C). Although an apparent increase in CagA levels was observed (Figure 1C), densitometric analysis revealed that CagA^PY^ levels remain relatively stable throughout the infection period (Figure 1D). The fluctuations observed in CagA^PY^ may be explained by the dynamic phosphorylation and dephosphorylation processes that CagA undergoes once inside epithelial cells. In early stages of infection, Src kinases phosphorylate the EPIYA motifs; however, these enzymes are eventually downregulated and inactivated. In contrast, during prolonged infection, Abl kinase contributes to sustained CagA phosphorylation. Additionally, phosphatases such as SHP1 can dephosphorylate the EPIYA motifs. These reversible modifications result in multiple phosphorylation states of CagA (Mueller et al., 2012; Hatakeyama, 2017).

Immunofluorescence analysis corroborated the presence of CagA in *H. pylori* CagA^+^ BxPC-3-infected cells in both the membrane and the cytoplasm; CagA was not noticed in BxPC-3 cells infected with the CagA^−^ *H. pylori* strain (Figure 1E).

### 3.2 CagA reduces TEER in pancreatic cells

**FIGURE 2:**
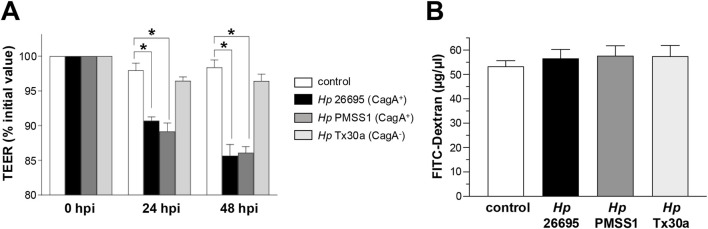
Integrity of the epithelial barrier of BxPC-3 cells. Pancreatic BxPC-3 cells were cultured on Transwell filters until confluence, and development of stable TEER was achieved. Cells were then infected apically with different *Helicobacter pylori* strains. **(A)** TEER was monitored over a 48-h period. Data were normalized by setting the initial TEER value (∼300 Ω cm^2^) as 100% for each well. **(B)** Paracellular permeability was assessed after 24 hpi using FITC-conjugated dextran. Fluorescence in the basolateral compartment was measured to quantify macromolecular flux. Statistical analysis was performed using Student’s t-test with a Bonferroni correction. (*) *p* < 0.05.

To evaluate the potential effect of CagA on barrier function, we analyzed the regulation of ion and macromolecule fluxes. Infection with CagA^+^ strains (26695 and PMSS1) induced 10% and 15% decreases in TEER at both 24 and 48 hpi, respectively, compared to the CagA^−^ strain (Tx30a) and uninfected controls, which maintained stable barrier function (Figure 2A).

To assess BxPC-3 monolayer permeability, FITC-conjugated dextran experiments were performed. The results showed that CagA internalization in BxPC-3 cells did not alter dextran flux, indicating a similar permeability level compared to both CagA-infected and uninfected control cells (Figure 2B).

These data suggest that, in pancreatic epithelial cells, infection with CagA^+^ *H. pylori* strains increases ion flux without affecting the macromolecular permeability, indicating a selective disruption of tight junction protein function and the possible involvement of specific ones.

### 3.3 CagA affects the amount of intercellular junction proteins in BxPC-3 pancreatic cells

*H. pylori* can use the effector protein CagA to interact with intercellular junctions, thereby disrupting the organization and function of the AJC in epithelial cells (Stein et al., 2013). To elucidate the mechanisms underlying barrier disruption, we analyzed the expression levels of various AJC components in pancreatic epithelial cells infected with either CagA^+^ or CagA^−^ *H. pylori* strains.

**FIGURE 3:**
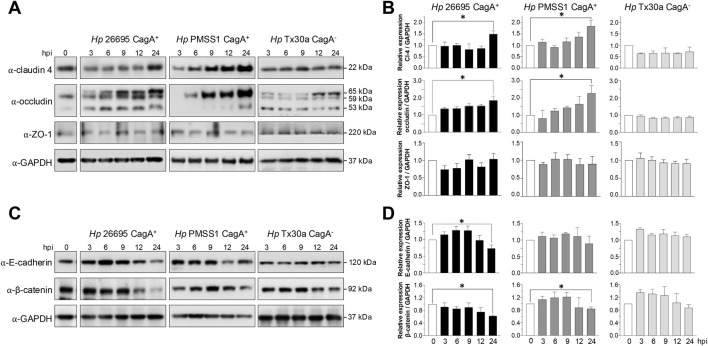
Amount of TJ and AJ proteins of BxPC-3 cells infected with H*. pylori*. Pancreatic cells were infected with *Hp* CagA^+^ and CagA^−^ strains at MOI 1:100. Cellular protein extracts were collected at 3, 6, 9, 12, and 24 hpi and analyzed by Western blot using antibodies against TJ and AJ proteins. GAPDH was used as a loading control. **(A)** Immunodetection of TJ proteins (claudin-4, occludin, and ZO-1). **(B)** Densitometric quantification of TJ proteins normalized to GAPDH. **(C)** Immunodetection of AJ proteins (E-cadherin and β-catenin). **(D)** Densitometric quantification of AJ proteins normalized to GAPDH. Molecular weights (in kDa) are indicated to the right of each blot. Statistical analysis was performed using Student’s t-test with a Bonferroni correction. (*) *p* < 0.05.

BxPC-3 pancreatic cells were harvested at 3, 6, 9, 12, and 24 hpi to obtain protein lysates. Western blot analyses were performed using specific antibodies against claudin-4, occludin, ZO-1, E-cadherin, and β-catenin. In cells infected with CagA^+^ strains, claudin-4 levels tended to increase, reaching statistical significance at 24 hpi (Figure 3A). No changes were observed in cells infected with CagA-deficient *H. pylori* strains (Figure 3B). Occludin showed a similar expression pattern to that of claudin-4. In BxPC-3 cells infected with the 26695 strain, the occludin level was elevated at all time points analyzed; and a significant increase was observed at 24 hpi with the 26695 and PMSS1 strains. Moreover, the bands detected using the occludin-specific antibody differed between epithelial cells infected with the two strains (Figure 3B). Occludin undergoes various post-transcriptional and post-translational modifications (including proteolysis, phosphorylation, dimerization, and ubiquitination), which result in distinct molecular weights and have diverse implications for TJ assembly and barrier function (Cummins, 2012). In Caco-2 cells infected with *H. pylori*, a low-molecular-weight form has been reported, suggesting that occludin turnover is rapidly disrupted in the presence of the bacterium. The authors proposed that ammonium, a byproduct of *H. pylori* urease activity, imposes additional stress on epithelial integrity (Lytton et al., 2005). Furthermore, *H. pylori* infection of AGS epithelial cells induces occludin expression in a CagA-dependent manner (Guillemin et al., 2002). Thus, the differential levels of CagA in the 26695 and PMSS1 strains could differentially affect occludin at the post-transcriptional and post-translational levels; however, further experiments are required to confirm this. In contrast to what is observed in gastric epithelial cells, no changes in ZO-1 protein levels were detected in pancreatic epithelial cells infected with either CagA^+^ or CagA^−^ strains (Figures 3A,B).

Regarding AJ components, both E-cadherin and β-catenin levels were reduced at 24 hpi in cells infected with CagA^+^ strains but remained unchanged in cells infected with the CagA^−^ strain (Tx30a) (Figures 3C,D).

### 3.4 CagA modifies the localization of intercellular junction proteins in pancreatic cells

**FIGURE 4:**
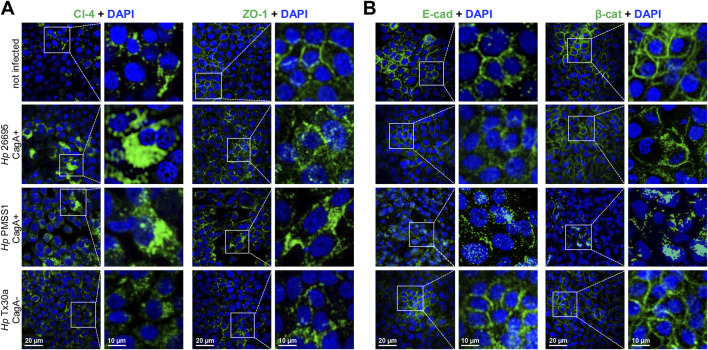
Localization of TJ and AJ proteins in BxPC-3 cells infected by *H. pylori*. Pancreatic cells were infected with different bacterial strains during 24 h and then processed using immunofluorescence assays, with specific antibodies against TJ **(A)** and AJ **(B)** proteins. Nuclei were counterstained with DAPI. Selected regions are shown at higher magnification in the right panels.

To investigate the impact of *H. pylori* infection on junctional integrity, the localization of intercellular junction proteins was examined in infected BxPC-3 pancreatic cells by immunofluorescence experiments, using specific antibodies. In non-infected cells, claudin-4, ZO-1, E-cadherin, and β-catenin displayed their typical localization at the cell borders. However, infection with CagA^+^ strains resulted in marked disruption of this pattern (Figure 4). Intercellular junction proteins were internalized toward the cytoplasm, accompanied by an apparent increase in claudin-4 and ZO-1 levels (Figure 4A), along with a decrease in E-cadherin and β-catenin levels (Figure 4B). These observations were consistent with the Western blot results, except for ZO-1, where no significant change in protein abundance was detected (Figure 3). Surprisingly, infection with the CagA^−^ strain (Tx30a) also induced mislocalization of these proteins from the membrane to the cytoplasm, although the effect was notably less pronounced compared to CagA^+^ strains (Figure 4).

These findings suggest that CagA contributes to the disruption of both the localization and expression of certain TJ and AJ proteins in pancreatic cells, potentially compromising epithelial barrier integrity and function, as previously reported in gastric epithelial models (Maria et al., 2013).

### 3.5 CagA alters the localization but not the level of actin in pancreatic cells

**FIGURE 5:**
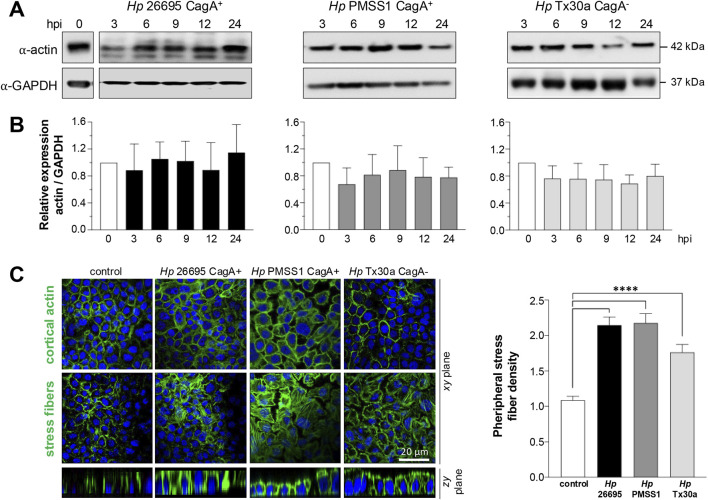
Amount and localization of actin in BxPC-3 cells infected with *H. pylori*. Pancreatic cells were infected with CagA^+^ and CagA^−^ *Helicobacter pylori* strains at MOI 1:100. **(A)** Protein lysates were collected at 3, 6, 9, 12, and 24 hpi and analyzed using Western blot experiments with an antibody against actin. GAPDH was used as a loading control. Molecular weights (kDa) are indicated to the right of each blot. **(B)** Densitometric quantification of actin levels normalized to GAPDH. Data are presented as mean ± standard error (SEM) from at least three independent experiments. Statistical significance was determined using Student’s t-test with a Bonferroni correction. **(C)** Pancreatic cells infected for 24 hpi were processed for fluorescence staining using FITC–phalloidin to label actin (green), and nuclei were counterstained with DAPI (blue). Confocal microscopy images were acquired at the apical (top) and basal (bottom) regions of *xy*-planes to visualize the cortical actin ring and stress fiber formation, respectively. In addition, *zy*-planes of each field were also included. The density of peripheral stress fibers was quantified in 100 cells per condition and normalized against the control group. Bar = 20 μm.

Bacterial factors interact with the host cell actin cytoskeleton to facilitate changes in its morphology and promote the *H. pylori* invasion and migration within the gastric mucosa (Selbach et al., 2003). To assess the impact of CagA on the actin cytoskeleton of pancreatic cells, we analyzed both the amount and subcellular localization of actin in BxPC-3 cells infected with *H. pylori* CagA^+^ and CagA^–^ strains. Western blot analysis revealed no significant changes in total actin levels at any of the time points tested compared to uninfected control cells (Figures 5A,B).

Nevertheless, the subcellular distribution of actin, particularly at the cortical region and in stress fibers, was markedly altered by infection (Figure 5C). These findings suggest that CagA may modulate the actin cytoskeleton in BxPC-3 pancreatic cells to influence epithelial cell motility.

### 3.6 Cortactin and vinculin expression and localization are differentially affected by *Helicobacter pylori* infection in pancreatic cells

**FIGURE 6:**
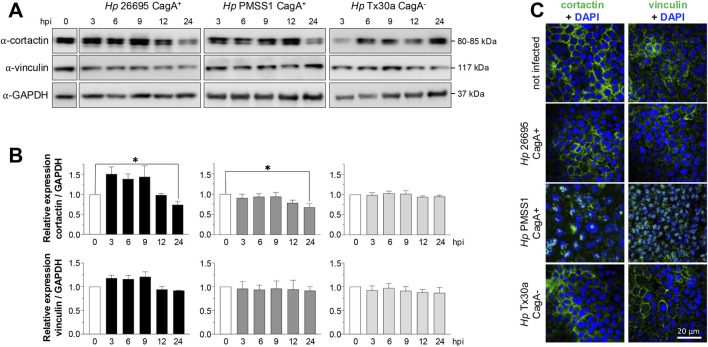
Amount and localization of cortactin and vinculin in BxPC-3 cells infected with *H. pylori*. Pancreatic cells were infected with CagA^+^ and CagA^−^ *Helicobacter pylori* strains at MOI 1:100. **(A)** Protein lysates were collected at 3, 6, 9, 12, and 24 hpi and analyzed using Western blot experiments with antibodies against cortactin and vinculin. GAPDH was used as a loading control. Molecular weights (kDa) are indicated to the right of each blot. **(B)** Densitometric quantification of cortactin and vinculin levels normalized to GAPDH. Data are presented as mean ± standard error (SEM) from at least three independent experiments. Statistical significance was determined using Student’s t-test with a Bonferroni correction. (*) *p* < 0.05. **(C)** Pancreatic cells infected for 24 hpi were processed for immunofluorescence staining using specific antibodies against cortactin and vinculin (green), and nuclei were counterstained with DAPI (blue). Bar = 20 μm.

Actin dynamics are regulated by a variety of associated proteins, including cortactin and vinculin. The Western blot analysis of BxPC-3 cells infected with the CagA^+^ 26695 strain demonstrated that cortactin levels tend to increase at 3 and 6 hpi, followed by a significant decrease at 24 hpi (Figures 6A,B). A similar pattern was observed in cells infected with the CagA^+^ PMSS1 strain, with cortactin expression decreasing notably at 24 hpi. In contrast, cortactin levels remained stable over time in cells infected with the CagA^–^ Tx30a strain (Figures 6A,B).

Contrasting to cortactin and similar to actin, vinculin expression remained unchanged at all time points and under all infection conditions (Figures 6A,B). Consistent with actin, the subcellular localization of cortactin and vinculin was also affected by infection. Both proteins were redistributed from the plasma membrane toward the nuclei, particularly in cells infected with the PMSS1 strain. This effect was less pronounced in cells infected with the 26695 CagA^+^ and Tx30a CagA^–^ strains (Figure 6C).

### 3.7 CagA affects the migration of pancreatic cells

**FIGURE 7:**
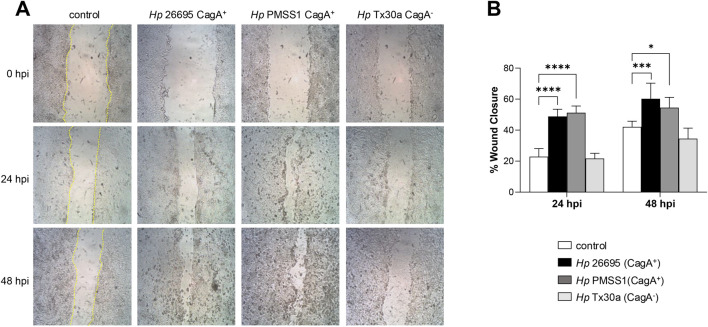
Migration of pancreatic cells infected with *H. pylori*. Confluent BxPC-3 cells were wounded using a pipette tip and then infected with *Helicobacter pylori* CagA^+^ and CagA^−^ strains at an MOI of 100 for 48 h. **(A)** The wound closure area was measured at 24 and 48 hpi. The scratch area was drawn in yellow lines in the control cells. **(B)** The percentage of wound closure was statistically analyzed using Student’s t-test with a Bonferroni correction. Data are presented as mean ± SEM (n = 3). (*) *p* < 0.05; (***) *p* < 0.001; (****) *p* < 0.0001.

Dynamic rearrangement of the actin cytoskeleton is a key hallmark of *H. pylori*-infected gastric epithelial cells, promoting cell migration and invasive growth (Wessler et al., 2011). To assess whether *H. pylori* infection similarly affects the BxPC-3 pancreatic cell motility, we performed a wound healing assay. Cell migration into the wound area was monitored microscopically at 24 and 48 hpi. Cells infected with the CagA^+^ strains exhibited significantly enhanced motility, closing the wound gap more rapidly than either uninfected controls or cells infected with CagA^−^ strains (Figure 7). These results suggest that CagA contributes to the stimulation of pancreatic cell motility.

### 3.8 CagA induces IL-8 production in infected pancreatic cells

**FIGURE 8:**
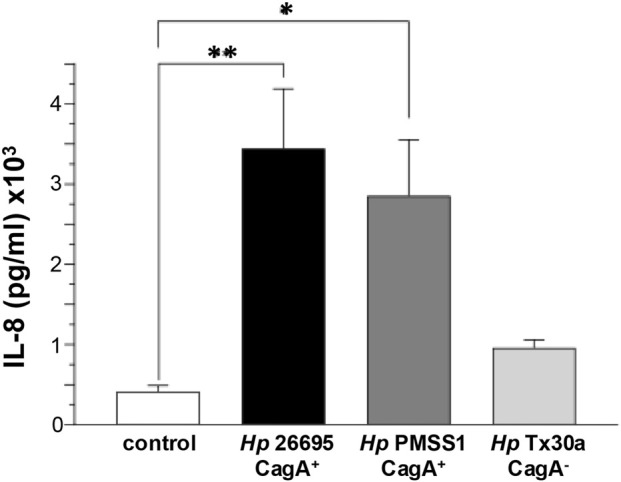
IL-8 secretion by pancreatic cells infected with *H. pylori*. BxPC-3 cells were infected with different *Helicobacter pylori* strains, and IL-8 levels were quantified from the culture supernatant using an ELISA assay. Data are presented as mean ± standard error (SEM) and were statistically analyzed using Student’s t-test with a Bonferroni correction. (*) *p* < 0.05; (**) *p* < 0.01.

CagA stimulates the secretion of proinflammatory cytokines, notably IL-8, by epithelial cells (Ferreira et al., 2016). To assess this effect in pancreatic cells, we measured IL-8 levels in the supernatant of BxPC-3 cells infected with either CagA^+^ or CagA^−^ *H. pylori* strains. Our results revealed a significant increase in IL-8 secretion, particularly in cells infected with CagA^+^ strains. A modest elevation was also observed in cells infected with the CagA^−^ strain compared to uninfected controls (Figure 8). These findings suggest that, as in gastric epithelial cells, CagA plays a role in promoting inflammatory responses in pancreatic cells, although other *H. pylori* virulence factors may also be involved in IL-8 production.

## 4 Discussion

Infections caused by CagA^+^ strains are associated with more severe gastric mucosal inflammation, advanced atrophic gastritis, and a significantly increased risk of developing gastric cancer (Takahashi-Kanemitsu et al., 2020). Moreover, this strain is associated to various extragastric diseases, including an elevated risk of ischemic stroke, coronary artery disease, and pancreatic cancer (Ai et al., 2015; Gravina et al., 2018).

The role of *H. pylori* infection in non-gastric tissues has been poorly investigated, and its effects and underlying mechanisms in pancreatic cells remain largely unexplored. Therefore, in this study, we evaluated the impact of the CagA virulence factor using different *H. pylori* strains, including CagA^+^ and CagA^−^, in a pancreatic ductal adenocarcinoma cell line. The CagA^+^ strain 26695, obtained from ATCC, serves as a reference strain and contains all canonical *H. pylori* virulence factors. PMSS1 CagA^+^, for its part, is a genetically engineered strain designed to enhance infectivity in mouse models. It carries multiple copies of the *cagA* gene and exhibits a virulence profile comparable to that of the 26695 strain (Jang et al., 2017; Kurniasari et al., 2023). In contrast, the CagA^−^ strain lacks the *cag*PAI and therefore does not express *cagA* (Pillinger et al., 2007).

In gastric epithelial cell lines such as AGS and MKN45, CagA exerts similar effects upon successful translocation into host cells and subsequent tyrosine phosphorylation at EPIYA motifs located in its C-terminal region. This phosphorylation is mediated by host kinases of the Src and Abl families (M. Krisch Linda et al., 2016; Tegtmeyer et al., 2020). These prior studies employed *H. pylori* strains including NCTC11637, 26695, Ka88, N6, P1, G27, and P12 (Asahi et al., 2000; Mueller et al., 2012; Knorr et al., 2021). Accordingly, our results showed that only the CagA^+^ strains were capable of translocating CagA into pancreatic cells, where it underwent tyrosine phosphorylation (Figure 1), potentially contributing alongside other virulence factors, to epithelial injury and the pathophysiology of *H. pylori* infection beyond the stomach.

*H. pylori* employs a wide range of strategies to disrupt the epithelial barrier, altering the distribution of AJC, inducing DNA damage, modulating apoptosis, affecting proliferation, migration, and invasion, stimulating cytokine production, and promoting cellular transformation (Alzahrani et al., 2014). In our study, infection with CagA^+^ *H. pylori* strains led to a marked reduction in the TEER of pancreatic epithelial cells, approximately 10% and 15% at 24 and 48 hpi (Figure 2A). These results are consistent with findings in gastric epithelial cell lines (MKN28 and NCI-N87), where infection with *H. pylori* strains 60190 and 26695 caused a progressive, time-dependent decrease in TEER, reaching reductions of 39% and 62% at 24 and 48 hpi, respectively, compared to uninfected monolayers (Wroblewski et al., 2009; Maria et al., 2013). Similarly, in gastroid monolayers infected with the *cag*PAI-positive *H. pylori* strain TN2GF4 at MOI 1:100, TEER decreased by 51% relative to uninfected controls (Uotani et al., 2019). The effect of *H. pylori* on pancreatic cells is less pronounced than on gastric cells as the pancreatic epithelium does not form a tight barrier and expresses fewer AJC components than the gastric epithelium, where permeability is more restricted (Guttman and Finlay, 2009; Kojima et al., 2013). Nevertheless, CagA does not appear to affect macromolecular flux (Figure 2B). This contrasts with findings in SCBN intestinal epithelial monolayers exposed to *H. pylori* SS1, where a significant increase in the permeability of 3,000 MW dextran was observed (Fedwick et al., 2005).

In gastric epithelial cells, the oncoprotein CagA induces the delocalization of intercellular junction proteins such as ZO-1, E-cadherin, and β-catenin, which leads to epithelial barrier impairment (Alzahrani et al., 2014). In this study, we observed the elevated levels of both claudin-4 and occludin during infection with *CagA* ^*+*^ *H. pylori* strains. Consistently, claudin-4 was relocated from the cell borders in uninfected cells to the cytoplasm in *CagA* ^*+*^ *H. pylori*-infected cells, suggesting functional impairment (Figure 3). Claudin-4 is a major component of TJ, involved in regulating the paracellular ion flux and maintaining cell polarity. Notably, this claudin is overexpressed in several malignancies, including breast, pancreatic, prostate, and ovarian cancers, where it contributes to increased cell invasion, motility, and survival (Hou, 2019). Conversely, in primary human gastric epithelial cells, infection with the *H. pylori* P1 strain did not alter the expression levels of ZO-1 and p120ctn, although their localization was disrupted, with signals observed in the nucleus and at the leading edge of migrating cells (Krueger et al., 2007). The lack of significant alterations in ZO-1 expression is consistent with our results, which showed high variability in infected cells, regardless of the strain used (Figure 3). Although CagA may affect ZO-1 binding and destabilize tight junctions (Amieva et al., 2003; Krueger et al., 2007), additional experiments would be required to demonstrate significant differences in the pancreas. The impact on the epithelial barrier was assessed by immunofluorescence, where ZO-1 was observed to be delocalized from the cellular borders toward the cytoplasm (Figure 4A).

Overall, the effect of *H. pylori* through CagA appears to be tissue-specific, possibly due to differential expression of AJC components. The gastric epithelium forms a tighter barrier, thanks to the presence of more AJC proteins with a higher expression, such as claudin-1, ZO-1, occludin, E-cadherin, and β-catenin, compared to the pancreas, where barrier permeability is greater to facilitate secretion because of the expressions of claudin-4, tricellulin, E-cadherin, and β-catenin (Kojima et al., 2013; Kyuno et al., 2021).

CagA interacts with E-cadherin in a tyrosine phosphorylation-independent manner, destabilizing the E-cadherin/β-catenin complex and resulting in cytoplasmic and nuclear accumulation of β-catenin (Murata-Kamiya et al., 2007). In our study, infection of pancreatic cells with the 26695 and PMSS1 strains led to a reduction in E-cadherin and β-catenin protein levels (Figure 3), accompanied by their redistribution to the cytoplasm (Figure 4). Similarly, Marques et al. (2018) reported that infection with the *H. pylori* 26695 strain significantly reduced E-cadherin, β-catenin, occludin, and ZO-1 protein levels in both MKN-74 and NCI-N87 cell lines and also caused delocalization of these junctional proteins (Marques et al., 2018).

In addition, CagA induces structural changes involving the rearrangement of the host actin cytoskeleton, leading to cell elongation, a phenomenon commonly referred to as the hummingbird phenotype (Tohidpour and Gorrell, 2017; Sain et al., 2025). Both membrane tethering of CagA and activation of SHP-2 (Src homology 2 domain-containing protein tyrosine phosphatase) are required to trigger this phenotype, which has been observed in gastric epithelial cell lines such as AGS and MKN-45. However, in other cell lines, including Caco-2, AZ-521, and MDCK, the hummingbird phenotype is not apparent; instead, the observed changes resemble an epithelial-to-mesenchymal transition (Bagnoli et al., 2005; Tegtmeyer et al., 2019). This variation may be attributed to the involvement of CEACAM receptors and their bacterial ligand HopQ, which are essential for full T4SS functionality (Bonsor et al., 2018). Therefore, the injection and phosphorylation of CagA may be cell line-specific, depending on the differential expression of CEACAM receptors. Notably, in the present study, significant morphological changes were observed in BxPC-3 pancreatic cells, consistent with epithelial transformation. This work is, therefore, relevant as it represents the first report evaluating the role of CagA in the pancreas and its molecular impact.

Moreover, these processes encompass a wide range of actin-binding proteins that contribute to actin stabilization, bundling, and branching, such as cortactin, vinculin, ezrin, and vimentin (Wessler et al., 2011). In this study, we observed that infection with the *H. pylori* PMSS1 strain in pancreatic cells led to constriction of the cortical actin ring. Furthermore, we noted increased formation of actin stress fibers (Figure 5). Actin, together with myosin, forms a dense perijunctional ring at the level of AJs and TJs. Actomyosin contraction is regulated by the phosphorylation of the myosin II regulatory light chain (MLC). MLC kinase (MLCK) promotes reorganization of perijunctional F-actin and increases epithelial permeability by redistributing TJ-associated proteins such as ZO-1, occludin, and claudins (Zolotarevsky et al., 2002; Wang et al., 2005). Therefore, changes in F-actin organization can enhance epithelial permeability via MLC phosphorylation and the subsequent remodeling of TJ proteins. Our findings are consistent with those of previous studies in AGS cells infected with the *H. pylori* P1 strain, where cytoskeletal rearrangements were distinguished (Selbach et al., 2003), and with observations of enhanced stress fiber formation in AGS cells infected with the *H. pylori* LC11 strain (Su et al., 2003).

Cortactin is involved in several cellular processes, including adhesion, cytoskeletal dynamics, cell motility, and epithelial–mesenchymal transition (EMT) (Tomar et al., 2012). Vinculin, meanwhile, links the actin cytoskeleton to cell adhesion complexes, both at focal adhesions (cell–substrate contacts) and AJs (cell–cell contacts) (James et al., 2023). In this study, infection with CagA^+^ *H. pylori* led to a decrease in cortactin levels, but not vinculin, in BxPC-3 cells (Figure 6). However, both proteins showed altered subcellular localization, shifting from the cell membrane to the cytoplasm (Figure 6), suggesting that their functional activity may be impaired by CagA. In contrast, cortactin was overexpressed in AGS and Caco-2 epithelial cells infected with *H. pylori cag*PAI-positive strains such as NCTC11637, P12, 26695, and G27 (Sharafutdinov et al., 2021). Vinculin levels, by comparison, remained constant in AGS cells infected with the P1 strain across all time points (30 min–24 hpi) (Moese et al., 2007).

Vinculin plays an essential role in recruiting the Arp2/3 complex, while cortactin stabilizes it at branched actin filaments, especially at AJs and focal adhesions, thereby contributing to major cytoskeletal remodeling (James et al., 2023). Through their association with Arp2/3, cortactin and vinculin may play functional roles in both cell–substrate and cell–cell adhesion, thereby influencing migration and invasion (van Rossum et al., 2006). CagA upregulates cortactin expression via the c-Jun N-terminal kinase (JNK) pathway, which inhibits Snail1, a regulator of the *cttn* gene, and promotes cortactin stabilization through paxillin and FAK, protecting it from degradation (Sharafutdinov et al., 2021). The wound-healing assays performed in this work demonstrated that infection with CagA^+^ strains increased pancreatic epithelial cell migration (Figure 7), aligning with previous findings in AGS cells infected with *H. pylori* NCTC1167 (Kikuchi et al., 2012).

Additionally, CagA manipulates cortactin phosphorylation. In particular, tyrosine residues Y412 and Y486 on cortactin become dephosphorylated by an unidentified phosphatase excluding SHP2, while serine phosphorylation at S405 and/or S418 is mediated via EGFR and ERK1/2 signaling (Knorr et al., 2021). This phosphorylation pattern promotes binding of cortactin’s SH3 domain to FAK, enhancing FAK kinase activity and host cell adhesion, mechanisms that prevent cell detachment in the gastric epithelium (Tomar et al., 2012). As a result, FAK and the associated tyrosine kinases Src and Abl become fully activated, leading to sustained phosphorylation of CagA and the detachment of cortactin from its membrane-associated pattern. The redistribution of cortactin to the cytoplasm can ultimately drive cytoskeletal rearrangements, increased cell motility, scattering, and elongation (Knorr et al., 2021). In addition, owing to the inactivation of c-Src via CagA interaction, vinculin is dephosphorylated at tyrosine residues, 100 and 1065, by corresponding phosphatases. Vinculin dephosphorylation disturbs the interaction and recruitment of the actin-related protein 2/3 (Arp2/3) complex by p34Arc, resulting in a reduction in focal adhesion complexes (Moese et al., 2007). *H. pylori* may act on vinculin in a similar manner as it does on cortactin, destabilizing its interaction with the Arp2/3 complex at the cell periphery and thereby contributing to cytoskeletal remodeling.

CagA also stimulates proinflammatory cytokine secretion, including IL-8, through activation of the NF-κB pathway in epithelial cells. IL-8 is a potent neutrophil chemoattractant and plays a critical role in the host’s inflammatory response (Fazeli et al., 2016; Ferreira et al., 2016). Consistent with this, our results show that IL-8 secretion was significantly higher in cells infected with CagA^+^ strains than in uninfected cells or those infected with the CagA^−^ strain (Figure 8). Previous studies have confirmed that IL-8 expression levels depend on the presence of CagA and the *cag*PAI locus (Fazeli et al., 2016; Uotani et al., 2019). Furthermore, cortactin has been identified as a key factor in maximal activation of NF-κB and subsequent IL-8 production during *H. pylori* infection (Tegtmeyer et al., 2022).

In summary, our findings highlight the involvement of the CagA oncoprotein in pathogenesis of the *H. pylori* infection beyond the gastric environment and suggest a potential role in the development of extragastric diseases. In particular, we found that CagA disrupts epithelial barrier integrity by altering intercellular junction proteins and induces actin cytoskeleton remodeling through the delocalization of actin, vinculin, and cortactin. These changes promote cell migration and an inflammatory response in pancreatic epithelial cells. Although we cannot rule out that the differences observed in this study may be attributed to the genetic variability among the *H. pylori* strains used, given their highly variable genomes, which include numerous gene rearrangements, inversions, sequence variations, and gene gains or losses, such genomic changes have even been reported to occur within the same strain during the course of infection. Strain 10700, now referred to as PMSS1 (pre-mouse SS1), is capable of long-term colonization in the mouse stomach and has become a widely used experimental model due to its strong ability to induce disease, largely owing to its expression of key virulence factors such as VacA and CagA (Draper et al., 2017). Strain 26695, which also contains CagA, is considered a Western-type strain and serves as a reference in many genetic and virulence studies (Linz and Schuster, 2007). In both strains, CagA carries the same EPIYA-A, EPIYA-B, and EPIYA-C phosphorylation motifs (Kurniasari et al., 2023). However, PMSS1 exhibits a heterogeneous population in terms of the *cagA* gene copy number, ranging from zero to four copies arranged as direct repeats within the chromosome (Jang et al., 2017). In contrast, strain Tx30a is a non-toxigenic isolate characterized by the *vacA s2/m2* genotype and the absence of the *cag*PAI. Unlike highly virulent strains, Tx30a does not produce the VacA cytotoxin and lacks cytotoxic activity in gastric epithelial cells, making it a useful reference strain for studying toxin variants and developing diagnostic and therapeutic tools (Letley et al., 2003). Thus, future studies should focus on elucidating the signaling pathways involved in these cellular events to better understand the role of CagA expressed by these *H. pylori* strains in pancreatic diseases such as pancreatitis, pancreatic cancer, and diabetes mellitus.

## Data Availability

The raw data supporting the conclusions of this article will be made available by the authors, without undue reservation.
